# Adherence to Biologic Therapies is Associated with Improved Clinical Outcomes and Reduced Healthcare Resource Utilization in Patients with Severe Asthma in Japan

**DOI:** 10.2147/JAA.S590261

**Published:** 2026-03-17

**Authors:** Hiroyuki Nagase, Urvee Karsanji, Hideaki Kunishige, Takeo Suzuki, Anna Vichiendilokkul, Liza Yuanita, Natsuki Sasakura, Jiaxuan Wang, Saeed Noibi

**Affiliations:** 1 Division of Respiratory Medicine and Allergology, Department of Medicine, Teikyo University School of Medicine, Tokyo, Japan; 2 Real World Biostatistics, GSK, London, UK; 3 Global Real-World Evidence & Health Outcomes Research, GSK, Tokyo, Japan; 4 Real World Data Analytics, GSK, Tokyo, Japan; 5 Global Medical Affairs, GSK, Collegeville, PA, USA; 6 Japan Medical Affairs, GSK, Tokyo, Japan; 7 Cytel Inc., Vancouver, BC, Canada; 8 Global Real-World Evidence & Health Outcomes Research, GSK, Jeddah, Saudi Arabia

**Keywords:** Asthma biologics, biologic adherence, cost analysis, exacerbations, group-based trajectory modeling, healthcare resource utilization

## Abstract

**Purpose:**

Biologic therapies have improved clinical outcomes and quality of life in patients with asthma, and treatment adherence is important for their effectiveness. This study evaluated 12-month adherence patterns for five asthma biologics approved in Japan and their impact on clinical and economic outcomes in patients with severe asthma.

**Methods:**

This non-interventional, cross-sectional, retrospective cohort study used Japan’s Medical Data Vision database (June 2009–September 2024). Adults with severe asthma, ≥30 days of continuous enrollment pre-biologic initiation and ≥12 months of follow-up were included. Adherence was assessed using medication possession ratio (MPR). Group-based trajectory modeling (GBTM) characterized distinct adherence trajectories over time, providing insights into heterogeneous adherence behaviors and subgroup patterns. Impact of biologic adherence on exacerbations (defined by hospital admissions, emergency department visits or requiring oral/intravenous corticosteroids), healthcare resource utilization (HCRU) and pharmacy costs were analyzed descriptively.

**Results:**

Among 2904 eligible patients, average MPR was 62.6%–72.6% across the five biologics. Over 90% of patients received ≥1 follow-up dose of their biologic; with average MPR increased by 1%–6% among these patients versus the overall cohort. The GBTM analysis was conducted in 2531 patients without a biologic switch during follow-up, identifying seven distinct clusters with MPR ranging from 10.0% to 94.8%. Patients were also classified as adherent (41.1%), partially adherent (28.1%), minimally adherent (3.2%), or treatment discontinuation (27.6%), based on dosing frequency and intervals. Mean exacerbation rates defined by hospital admissions were low (0.02–0.08 events per patient/year). Exacerbations of any type typically increased with declining biologic adherence. Decreased adherence was generally associated with increased HCRU and higher asthma-related pharmacy costs, particularly when biologic costs were excluded.

**Conclusion:**

Biologic adherence was consistently associated with fewer exacerbations, reduced HCRU and lower asthma-related pharmacy costs (excluding biologic costs), reinforcing the importance of optimizing adherence in patients with asthma.

## Plain Language Summary

Patients with severe asthma may have flare-ups (called asthma exacerbations), even when using standard treatments like inhaled steroids. For these patients, doctors may prescribe biologics which can reduce the number or severity of asthma exacerbations and are generally safer than long-term systemic corticosteroid use. However, biologics work best when taken as prescribed. This study investigated how well adults with severe asthma in Japan followed their prescribed biologic treatments (adherence to treatment). Also, the study investigated what the impact of adherence to treatment was on asthma exacerbations, need for healthcare support and pharmacy costs. The study researchers used health records from 2009 to 2024 to identify patients who started one of five biologics approved for asthma in Japan. Adherence was measured over 1 year by checking how often patients collected their biologic therapy (known as “medication possession ratio” [MPR]). Based on how often patients collected their medicine, they were categorized as adherent, partially adherent, minimally adherent, or treatment discontinuation (stopped collecting their medicine). The researchers also used a method called “group-based trajectory modeling” (GBTM) which identifies different patterns of missed doses over time. This identified seven different patterns of missed doses over time, including patients who stayed adherent, those who gradually collected their medicine less, and those who stopped collecting it for long periods. The number of asthma exacerbations, healthcare use, and pharmacy costs were then compared among patients in these different adherence groups. Patients who took their biologic as prescribed had fewer asthma exacerbations, needed less healthcare support, and had lower asthma-related pharmacy costs compared with those who did not. These results highlight the importance of taking biologic treatments consistently for patients with severe asthma to reduce the disease and economic burden.

## Graphical Abstract

**Figure:**
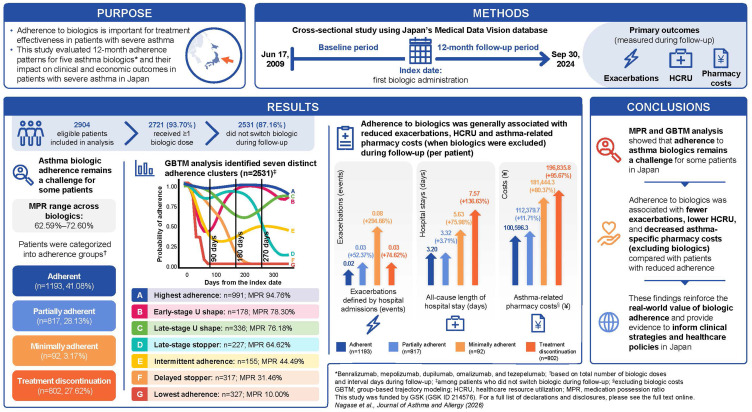

## Introduction

Asthma is a chronic inflammatory disease of the airways, characterized by recurrent episodes of airway constriction leading to symptoms of breathlessness, wheezing, and cough.^1^ Severe asthma affects 5–10% of the total asthma population worldwide,^2^ and accounts for approximately 2.4–12.7% of the asthma population in Japan.^3–6^ Severe asthma is associated with significant morbidity, mortality, impaired quality of life and substantial burden on patients and healthcare resources.^5^,^7^,^8^ Severe asthma is defined as asthma that remains uncontrolled despite optimized treatment with high-dose inhaled corticosteroids (ICS) combined with a long-acting beta-2-agonist (LABA), or that requires such treatments to maintain control.^1^ Some patients with severe asthma continue to experience uncontrolled symptoms despite receiving these treatments;^5^,^6^ for such patients, the addition of biologic therapy should be considered.^1^

Five biologic therapies are currently approved for use in severe asthma in Japan:^9^ omalizumab (anti-immunoglobulin E [IgE]), benralizumab (anti-interleukin [IL]-5 receptor), mepolizumab (anti-IL-5), dupilumab (anti-IL-4/13) and tezepelumab (anti-thymic stromal lymphopoietin).^10–14^ These biologics are prescribed in accordance with the Japanese Guidelines for Adult Asthma from the Japan Allergy Society, based on symptom control with existing inhaled therapies and phenotype as indicated by biomarkers such as allergen sensitization and blood eosinophil count.^15^ These biologic therapies have transformed asthma management, within Japan and globally, by reducing exacerbations, hospitalizations, and oral corticosteroids (OCS) use, and improving lung function, asthma control, and quality of life.^9^,^16^

Previous evidence suggests that adherence to asthma therapies including biologics is variable, in part due to methodological differences between studies, and that 5% to 63% of patients are either sub-optimally adherent or non-adherent.^17^ Studies conducted in Japan and in other countries have associated non-adherence to asthma medications with a range of practical, social and psychological factors, including access to specialist care, employment (active working) status, fear of injections, adherence to previous medication, depressive symptoms, patient education, and forgetfulness or perceived inconvenience of medication administration.^17–20^ Non-adherence to asthma treatments can substantially impact treatment effectiveness, leading to poor clinical outcomes for patients and increased healthcare resource utilization (HCRU).^21^ For example, poor adherence to inhaler therapies in severe asthma has been associated with increased exacerbations, high OCS use, and reduced symptomatic control and quality of life.^21^ Similarly, patients with ulcerative colitis or rheumatoid arthritis in Japan who are less adherent to biologic treatment demonstrate increased frequency of HCRU, specifically more hospitalizations and higher healthcare costs compared with more adherent patients.^22^,^23^ However, there are currently limited data from large studies investigating rates of biologic adherence and the relationship between treatment adherence and clinical outcomes among patients with severe asthma. In a retrospective analysis of 189 patients with severe asthma from the USA, treatment with mepolizumab led to real-world improvements in HCRU and costs over 4 years, likely driven by reduced exacerbation rates.^24^ Furthermore, patients with severe asthma who stop biologic therapy may be at increased risk of poor asthma control and exacerbations, highlighting the importance of persistence with biologic therapy to sustain clinical benefits in this population.^25^

This cross-sectional study was conducted in a cohort of adults with severe asthma in Japan and aimed to assess 12-month adherence patterns for the five biologics currently approved for asthma in Japan, and to evaluate how these adherence patterns impact clinical outcomes, HCRU, and healthcare costs. Three different methods were used to evaluate adherence: the medication possession ratio (MPR), predefined adherence criteria, and group-based trajectory modeling (GBTM) analysis, an innovative method that incorporates both the timing and frequency of missed doses to identify distinct adherence patterns. Collectively, these approaches complement one another and provide a more comprehensive understanding of biologic treatment adherence and its impact on outcomes.

## Methods

### Study Design

This was a non-interventional, cross-sectional, retrospective cohort study (GSK ID 214576) using data from Japan’s Medical Data Vision (MDV) claims database. The MDV is a hospital-based database that captures all health claims and diagnosis and procedure data including biologics, dispensed at over 500 hospitals.

**Figure 1:**
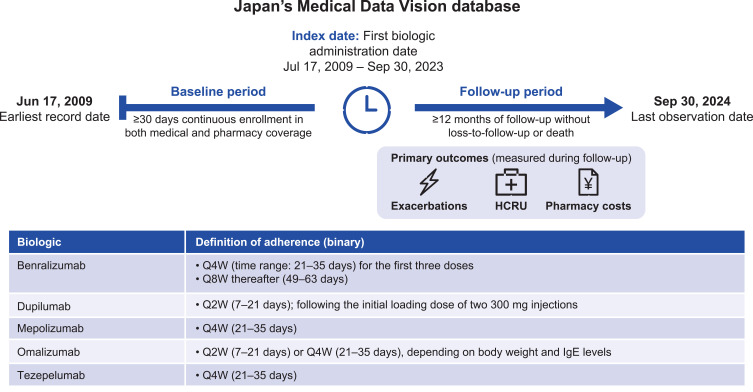
Study Design.

**Abbreviations**: IgE, immunoglobulin E; HCRU, healthcare resource utilization; QXW, every X weeks.

Adult patients with severe asthma in Japan who initiated biologic therapy were followed for the first 12 months post-initiation to allow for time-dependent changes in outcomes, HCRU, and healthcare costs and association with adherence (Figure 1). In Japan, biologic prescriptions for severe asthma are typically issued by respiratory specialists or allergy specialists and are dispensed via in-hospital pharmacies or external pharmacies. The index date was defined as the date of initiation of one of five biologics approved for asthma in Japan (benralizumab, dupilumab, mepolizumab, omalizumab or tezepelumab), and was required to fall between July 17, 2009, and September 30, 2023.

### Patient Population

Eligible patients were ≥18 years of age on the index date, with a diagnosis of asthma at any time prior to biologic initiation, identified from the database using disease codes designated by the Ministry of Health, Labor and Welfare of Japan. Patients were required to have initiated treatment with one of the five specified biologics as identified using Medical Data Vision healthcare receipt codes with ≥30 days of continuous enrollment prior to the index date, ≥12 months of follow-up without loss to follow-up or death, and no prior asthma biologic use. Additional inclusion criteria required patients to receive ≥1 prescription of both ICS and LABA within the 90 days prior to and including the asthma diagnosis date. ICS/LABA could be taken in combination with long-acting muscarinic antagonists (LAMA) if needed.

Patients were excluded if they had a prescription of multiple biologics on the index date, previous diagnosis of lung malignancies, active respiratory tuberculosis or cystic fibrosis any time before the index date, or diagnosis of hypereosinophilic syndrome or eosinophilic granulomatosis with polyangiitis any time before the end of the 12-month follow-up. Diagnosis of chronic spontaneous urticaria (for patients initiating dupilumab or omalizumab), atopic dermatitis (dupilumab), prurigo nodularis (dupilumab), seasonal allergic rhinitis (omalizumab), or chronic rhinosinusitis with nasal polyps (mepolizumab or dupilumab) at any time prior to the index date also prompted exclusion from the study.

### Assessment of Adherence

Adherence to each biologic during the follow-up was first assessed in a binary manner, with patients categorized as either adherent or non-adherent, based on prescription dates, adjustments for quantity per fill from the pharmacy claims dataset, and approved dosing schedules: benralizumab every 4 weeks (Q4W) for the first 3 doses, then every 8 weeks (Q8W); dupilumab every 2 weeks (Q2W); mepolizumab and tezepelumab Q4W; and omalizumab Q2W or Q4W depending on body weight and IgE level (Figure 1). A dynamic 7-day buffer before and after the desired treatment dose date was used to determine the adherence status (ie adherent or non-adherent), ensuring maximum capture of adherence data. The schedule for the next dose was then dynamically reset based on the date of the previous dose. The impact of adherence status, defined as periods during which patients received biologic treatment (adherent periods) versus periods during which they did not receive biologic treatment (non-adherent periods), was assessed using generalized linear mixed models and linear mixed-effects models.

The MPR was calculated for each biologic based on total number of days with medication possession, divided by total number of days in the 12-month follow-up period. Based on the number of missed doses and identified treatment gaps, patients were categorized into four treatment adherence groups: adherent, partially adherent, minimally adherent, and treatment discontinuation (Table 1). GBTM was assessed among patients who remained on the same biologic during the follow-up, and was used to capture the timing and frequency of missed doses to identify distinct longitudinal treatment-adherence clusters for patients with similar adherence patterns during the 12-month follow-up period. The GBTM clusters were presented across all five biologics collectively, and not stratified by biologic, to increase the sample size and identify generalizable adherence patterns representative of the overall severe asthma population in Japan.

**Table 1:** Categorization of Treatment Adherence for Each Biologic Over the 12-Month Follow-Up Period Based on Interval days and Number of Doses

	Adherent	Partially Adherent	Minimally Adherent	Treatment Discontinuation
**Benralizumab** Q4W for first 3 doses, then Q8W	All gaps within windows *AND* >4 doses Gap <56 days (between doses 1 and 2) Gap <84 days (between doses 2 and 3) Gap <112 days (after dose 3)	≥1 gap exceeds the recommended window with >4 doses *OR* ≤4 doses with all gaps less than recommended window Gap ≥56 days (between doses 1 and 2) Gap ≥84 days (between doses 2 and 3) Gap ≥112 days (after dose 3)	≥1 prolonged gap *AND* ≤4 total doses Gap ≥56 days (between doses 1 and 2) Gap ≥84 days (between doses 2 and 3) Gap ≥112 days (after dose 3)	Gap at the end of the 12 months, while remaining active in database Gap ≥112 days (between doses 1 and 2) Gap ≥140 days (between doses 2 and 3) Gap ≥168 days (after dose 3)
**Dupilumab** Q2W	<28-day gap between doses *AND ≥*13 doses	≥28-day gap with *≥*13 doses*OR* <13 doses with <28-day gap	<13 doses with ≥28-day gap	≥42-day gap at the end of 12 months, while still active in the database
**Mepolizumab, and tezepelumab** Q4W	<56-day gap *AND ≥*7 doses	≥56-day gap with *≥*7 doses *OR* <7 doses with <56-day gap	<7 doses with ≥56-day gap	≥84-day gap at the end of 12 months, while remaining active in database
**Omalizumab** Q2W or Q4W	Q2W: <28-day gap *AND ≥*13 doses Q4W: <56-day gap *AND ≥*7 doses	Q2W: ≥28-day gap with *≥*13 doses *OR* <13 doses with <28-day gap Q4W: ≥56-day gap with *≥*7 doses *OR* <7 doses with <56-day gap	Q2W: <13 doses with ≥28-day gap Q4W: <7 doses with ≥56-day gap	≥42-day gap (Q2W) or ≥84-day gap (Q4W) at end of 12 months, while remaining active in database

**Note**: If a patient met the criteria for both treatment discontinuation and any of the other adherence categories (adherent, partially adherent or minimally adherent) simultaneously, they were categorized in the treatment discontinuation group first. QXW, every X weeks.

### Outcomes

The study evaluated three primary outcomes during the follow-up across treatment adherence categories and clusters: exacerbations, HCRU, and associated pharmacy costs. Exacerbations were defined as events resulting in asthma-related hospital admissions, asthma-related emergency department (ED) visits or oral or intravenous (IV) corticosteroid administration. Both the frequency of exacerbation events and the proportion of patients experiencing exacerbation were reported.

The frequency and proportion of patients with all-cause and asthma-related HCRU were reported based on inpatient consultations, and included the number of hospital admissions, length of hospital stays, and the number of ED visits. The length of hospital stays included planned and unplanned hospitalizations. All-cause pharmacy costs include both outpatient and inpatient pharmacy costs, regardless of whether the costs were related to asthma or associated with hospitalization. Asthma-related pharmacy costs were those incurred between inpatient visits, with an asthma diagnosis falling between the admission and discharge dates. Both all-cause and asthma-related pharmacy costs were reported in Japanese Yen and were assessed including and excluding asthma biologic costs. Other assessed outcomes included baseline characteristics of the patient population, stratified by treatment adherence clusters.

Exploratory outcomes included the cumulative and average use of oral and IV corticosteroids during the follow-up, expressed in prednisone equivalents (mg). The cumulative amount was defined as the total sum of oral and IV corticosteroid exposure per patient, while the average amount was defined as the daily average oral and IV corticosteroid use per patient. The daily average was calculated only over the days that patients received OCS and IV steroids.

### Statistical Analysis

The sample size for the GBTM was determined based on the requirements for cluster identification, characterization, association analysis, and GBTM application. A minimum sample size of 450 patients was recommended to ensure that at least 50 patients were included per cluster, across an anticipated nine clusters.

Descriptive statistics were used to characterize baseline characteristics of the study population, treatment patterns, and outcome distributions. For continuous variables, means (standard deviations [SD]) and medians (interquartile range [IQR]) were calculated, while categorical variables were presented as the number and proportion of patients. Standardized mean differences (SMDs) were used to compare baseline characteristics across biologic treatment groups and adherence clusters. No imputation for missing data was made as, if no record exists, it is assumed the patient did not receive the treatment or service.

Statistical models were applied to evaluate the effect of treatment adherence on outcomes over the 12-month follow-up. To describe the association between adherence patterns and asthma-related outcomes, univariate Poisson generalized linear mixed models (GLMMs) were used for event count outcomes to investigate the effect of longitudinal binary treatment adherence on cumulative exacerbations and HCRU outcomes. Rate ratios were estimated to reflect the influence of adherence on event rates, and if overdispersion was detected, the models were switched to Negative Binomial GLMMs to account for extra variability. For cost outcomes, simple longitudinal regression using linear mixed-effects models was employed to assess the association between adherence and cumulative event-specific costs over 12 months, with effect sizes indicating the influence of adherence on average cost increases. For other continuous outcomes (eg pharmacy costs) with normal distributions, mean differences between clusters were evaluated using one-way analysis of variance; if normality was not met, the non-parametric Kruskal–Wallis test was applied. Count data (eg number of exacerbations) were analyzed using Poisson regression, or negative binomial regression if overdispersion was detected. Binary outcomes (eg the proportion of patients experiencing HCRU events) were assessed using logistic regression across adherence clusters. All analyses were conducted using R version 4.2.2 (R Foundation for Statistical Computing, Vienna, Austria).

### Ethical Approval and Patient Consent

This study was conducted as per the Declaration of Helsinki and the Ethical Guidelines for Medical and Health Research Involving Human Subjects. The protocol was reviewed and approved (Approval No. 210229) by the Non-Profit Organization MINS Institutional Review Board (Tokyo, Japan). The data for this study were extracted from a commercially available de-identified claims database. Accordingly, informed consent, ethics committee or institutional review board approvals were not required, as per the Act on the Protection of Personal Information and the Ethical Guidelines for Life Science and Medical Research Involving Human Subjects.^26^,^27^

## Results

### Patient Population

Overall, 2904 eligible patients were identified and included in the MPR analysis. A total of 1080 (37.19%) patients were prescribed benralizumab, 837 (28.82%) mepolizumab, 406 (13.98%) dupilumab, 375 (12.91%) omalizumab, and 152 (5.23%) tezepelumab.

Of the 2904 eligible patients, 2531 (87.16%) patients remained on the same biologic during the 12-month follow-up period and were included in the GBTM analysis. Of these, 953 (37.65%) received benralizumab, 706 (27.89%) mepolizumab, 402 (15.88%) dupilumab, 333 (13.16%) omalizumab, and 137 (5.41%) tezepelumab.

### Analysis of Treatment Adherence

Among the 2904 patients, the average MPR ranged from 62.59%–72.60% across the five biologics. Overall, 2721 (93.70%) patients received ≥1 follow-up dose of their biologic. The average MPR was 1.05%–5.81% higher in patients with ≥1 follow-up dose compared with the overall population. Treatment regimens were frequently interrupted and restarted with the same biologic and gaps in adherence were common, especially when treatments occurred outside the scheduled timeline, despite the use of the ±7-day buffer around the expected treatment date. Based on the total number of doses and the intervals between doses during the follow-up, patients were categorized as adherent (n=1193 [41.08%]), partially adherent (n=817 [28.13%]), minimally adherent (n=92 [3.17%]), or treatment discontinuation (n=802 [27.62%]).

### GBTM Analysis of Treatment Adherence

**Figure 2:**
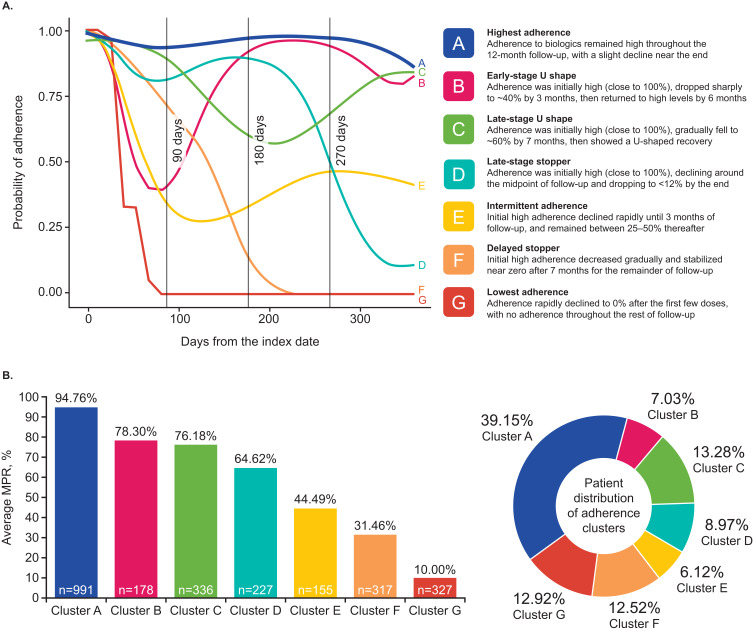
Adherence clusters identified through GBTM (**A**) and average MPR and patient distribution by GBTM cluster (**B**).

**Abbreviations**: GBTM, group-based trajectory modeling; MPR, medication possession ratio.

The GBTM analysis, evaluated in the 2531 patients who remained on the same biologic, identified seven distinct treatment adherence clusters with unique trajectories during the follow-up (Figure 2A and B). The clusters were arranged alphabetically from A–G, from highest to lowest adherence based on their average MPR: Cluster A – highest adherence (n=991 [39.15%]; MPR 94.76%); Cluster B – early-stage U shape (n=178 [7.03%]; MPR 78.30%); Cluster C – late-stage U shape (n=336 [13.28%]; MPR 76.18%); Cluster D – late-stage stopper (n=227 [8.97%]; MPR 64.62%); Cluster E – intermittent adherence (n=155 [6.12%]; MPR 44.49%); Cluster F – delayed stopper (n=317 [12.52%]; MPR 31.46%); and Cluster G – lowest adherence (n=327 [12.92%]; MPR 10.00%; Figure 2A and B).

Baseline characteristics were broadly balanced across GBTM clusters (SMD <0.30; Table 2). Median age, sex, and median Charlson Comorbidity Index was comparable between clusters. Smoking status exhibited moderate variability across clusters (SMD=0.177), with the proportion of current or former smokers ranging from 7.7% in Cluster E to 20.2% in Cluster G.

**Table 2:** Baseline Patient Characteristics Across GBTM Adherence Clusters (N=2531)

Covariate	Overall (N=2531)	Cluster A (N=991)	Cluster B (N=178)	Cluster C (N=336)	Cluster D (N=227)	Cluster E (N=155)	Cluster F (N=317)	Cluster G (N=327)	SMD
**Age, years, median (IQR)**	69.00 (55.00, 76.00)	70.00 (57.00, 76.00)	69.00 (56.00, 75.75)	68.00 (54.75, 75.00)	69.00 (55.50, 76.00)	69.00 (53.00, 78.00)	69.00 (53.00, 76.00)	71.00 (56.00, 78.00)	0.066
**Gender, %**									
Male	38.8	36.4	39.9	37.8	38.8	34.2	43.5	43.7	0.088
Female	61.2	63.6	60.1	62.2	61.2	65.8	56.5	56.3	
**Index year, median (min, max)**	2020 (2009, 2022)	2020 (2009, 2023)	2020 (2011, 2023)	2020 (2011, 2023)	2020 (2009, 2023)	2021 (2010, 2023)	2020 (2012, 2023)	2020 (2012, 2023)	0.088
**Smoking status, %**									
Current/Former	13.7	10.3	15.2	16.4	13.2	7.7	17.4	20.2	0.177
Never	23.6	24.1	23.0	19.6	23.8	25.2	22.1	26.9	
Unknown/Other	62.7	65.6	61.8	64.0	63.0	67.1	60.6	52.9	
**Charlson Comorbidity Index, median (min, max)**	4 (1, 16)	4 (1, 16)	4 (1, 13)	4 (1, 12)	4 (1, 11)	4 (1, 14)	4 (1, 15)	4 (1, 12)	0.065
**BMI, %^a^**									
Underweight^b^	4.9	3.0	3.9	4.8	6.2	3.2	6.0	9.8	0.198
Normal^c^	11.0	11.2	11.8	10.1	10.1	7.1	10.1	14.1	
Overweight^d^	14.4	15.1	11.8	12.2	16.3	12.9	16.4	13.1	
Obese^e^	9.1	8.2	11.2	10.1	7.0	10.3	8.5	11.3	
Unknown	60.6	62.5	61.2	62.8	60.4	66.5	59.0	51.7	
**BEC, cells/μL, median (IQR)^f^**	280.25 (95.80, 714.57)	491.15 (168.70, 843.98)	211.75 (30.16, 821.83)	260.00 (121.25, 713.21)	188.60 (94.75, 548.85)	101.73 (20.87, 704.90)	159.07 (77.80, 487.62)	188.54 (78.20, 375.36)	0.228
**Blood IgE level, IU/mL, median (IQR)**^g^	161.00 (41.20, 555.00)	241.20 (66.50, 703.25)	219.00 (82.55, 439.64)	149.00 (73.05, 728.00)	90.45 (31.52, 441.00)	127.50 (62.75, 223.23)	38.70 (13.20, 234.75)	216.00 (58.60, 639.00)	0.298

**Notes**: ^a^Among patients with known BMI: Overall, n=996 (39.4%); Cluster A, n=372 (37.5%); Cluster B, n=69 (38.8%); Cluster C, n=125 (37.2%); Cluster D, n=90 (39.6%); Cluster E, n=52 (33.5%); Cluster F, n=130 (41.0%); Cluster G, n=158 (48.3%); ^b^<18.5 kg/m^2; c^≥18.5 kg/m^2^ to <22.2 kg/m^2; d^≥22.2 kg/m^2^ to <26.9 kg/m^2; e^≥26.9 kg/m^2; f^among patients with ≥1 non-missing value for BEC: Overall, n=252 (9.96%); Cluster A, n=94 (9.49%); Cluster B, n=18 (10.11%); Cluster C, n=42 (12.50%); Cluster D, n=27 (11.89%); Cluster E, n=10 (6.45%); Cluster F, n=30 (9.46%); and Cluster G, n=31 (9.48%); ^g^among patients with ≥1 non-missing value for IgE: Overall, n=189 (7.47%); Cluster A, n=68 (6.86%); Cluster B, n=11 (6.18%); Cluster C, n=34 (10.12%); Cluster D, n=22 (9.69%); Cluster E, n=6 (3.87%); Cluster F, n=24 (7.57%); and Cluster G, n=24 (7.34%).

**Abbreviations**: BEC, blood eosinophil count; BMI, body mass index; GBTM, group-based trajectory modeling; IgE, immunoglobulin E; IQR, interquartile range; SMD, standardized mean difference.

### Exacerbations

**Figure 3:**
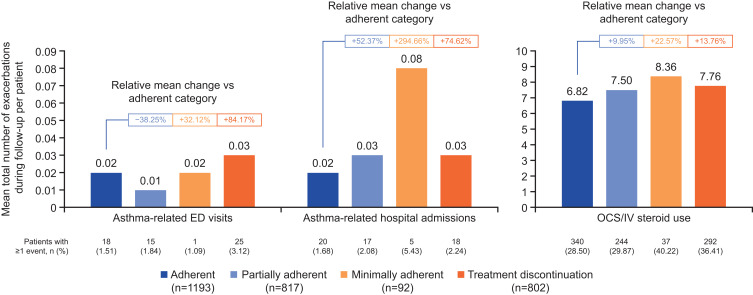
Summary of exacerbations (as defined by asthma-related ED visits, asthma-related hospital admissions or oral/IV corticosteroid use) by asthma biologic treatment adherence categories during the 12-month follow-up period (N=2904). Exacerbations requiring OCS/IV steroid use included OCS prescriptions lasting ≥3–≤28 days within the month of, or the month before asthma diagnosis, and any IV steroid used during or prior to the month of diagnosis.

**Abbreviations**: ED, emergency department; IV, intravenous; OCS, oral corticosteroid.

The regression analyses demonstrated that treatment adherence was associated with significantly reduced cumulative rates of asthma-related ED visits, hospital admissions and oral/IV corticosteroid use versus non-adherence (p<0.001; Supplementary Table 1). When assessed by adherence category, mean exacerbation rates defined by asthma-related ED visits or hospital admissions were low (0.01–0.08 events per patient; Figure 3). Exacerbation rates were lowest among patients in the adherent group across all categories, except for asthma-related ED visits, which were 38.25% lower among partially adherent patients (Figure 3). Compared with adherent patients, asthma-related ED visits were 32.12% higher in minimally adherent patients and 84.17% higher in treatment discontinuation patients. Exacerbations defined by hospital admissions were 52.37–294.66% higher among patients with reduced adherence versus those in the adherent group, with the greatest increase reported in patients with minimal adherence. Exacerbations requiring oral/IV corticosteroid use also increased by 9.95%–22.57% in patients with reduced adherence, relative to adherent patients.

When assessed by GBTM treatment adherence clusters, patients in Cluster A (highest adherence) demonstrated consistent improvements in exacerbation rates related to asthma-related ED visits, hospital admissions and oral/IV corticosteroid use compared with Cluster G (lowest adherence) during follow-up (Supplementary Figure 1). However, compared with Cluster A, the relative mean increase in the number of exacerbation events was highest in Cluster D (late-stage stopper) for exacerbations related to ED visits (118.28%) and requiring oral/IV corticosteroid use (81.59%), and Cluster F (delayed stopper) for exacerbations related to asthma-related hospital admissions (229.99%).

### HCRU

**Figure 4:**
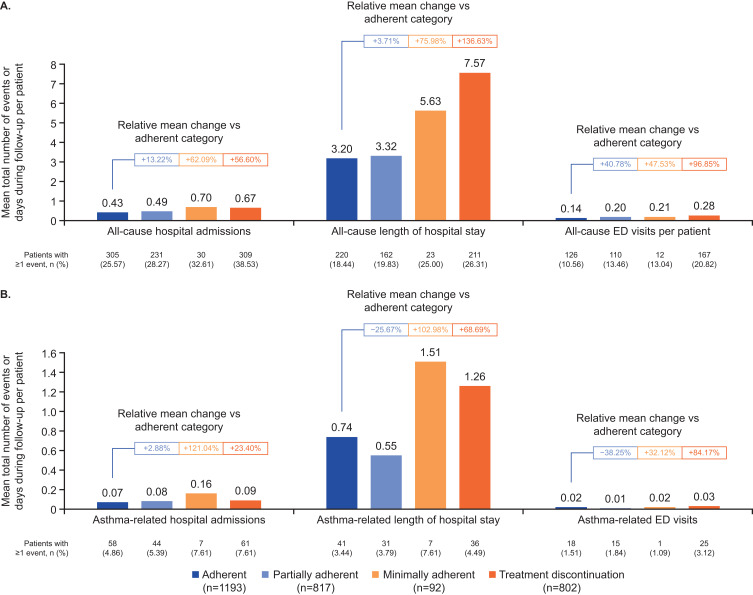
Summary of all-cause (**A**) and asthma-related (**B**) HCRU by adherence categories during the 12-month follow-up period (N=2904). All-cause hospital admissions included planned, unplanned and emergency hospitalization; length of hospital stays included planned and unplanned hospitalization; ED visits included emergency hospitalization only.

**Abbreviations**: ED, emergency department; HCRU, healthcare resource utilization.

Biologic adherence was consistently associated with significantly reduced rates of all-cause and asthma-related hospital admissions and ED visits compared with patients with reduced adherence (p<0.001), and resulted in a large reduction in length of hospital stay (Supplementary Table 1). HCRU analysis by adherence categories showed that adherent patients had the lowest rate of all-cause ED visits (10.56%; mean 0.14), while those who discontinued treatment had the highest (20.82%; mean 0.28), representing a relative mean increase of 96.85% (Figure 4A). All-cause hospital admissions and the length of all-cause hospital stays showed a similar trend. Asthma-related HCRU also followed a similar pattern but demonstrated greater variability (Figure 4B). Overall rates for asthma-related hospital admissions and ED visits remained low across groups (1.09%–7.61%) but rose steadily with decreasing adherence. The length of asthma-related hospital stays varied among groups; partially adherent patients had shorter stays (25.67% decrease relative to the adherent group) and minimally adherent patients had the longest (102.98% increase; Figure 4B).

The analysis of HCRU outcomes revealed substantial variability across GBTM adherence clusters. Patients with the highest adherence (Cluster A) consistently had the lowest all-cause HCRU across all measures, while patients in lower adherence clusters (Clusters D [late-stage stopper], F [delayed stopper] and G [lowest adherence]) tended to have higher levels of HCRU (Supplementary Figure 2A and B). Overall, 22.10% of patients in Cluster A (highest adherence) had ≥1 all-cause hospital admission compared with 31.61%–40.37% in Clusters D–G (late-stage stopper, intermittent adherence, delayed stopper, lowest adherence), and all-cause ED visits occurred in 8.61% of patients in Cluster A and 12.91–21.41% in Clusters D–G. Mean all-cause length of hospital stays was also lowest in Cluster A (highest adherence; 2.57 days) compared with 4.06–9.68 days in Clusters D–G (late-stage stopper, intermittent adherence, delayed stopper, lowest adherence). Greater variability was observed with asthma-related HCRU outcomes; compared with Cluster A, asthma-related hospital admissions, ED visits and the length of hospital stays were lowest in Cluster E (intermittent adherence), while Clusters D (late-stage stopper), F (delayed stopper) and G (lowest adherence) had the highest numbers of these events and longer hospital stays.

### Pharmacy Costs

**Figure 5:**
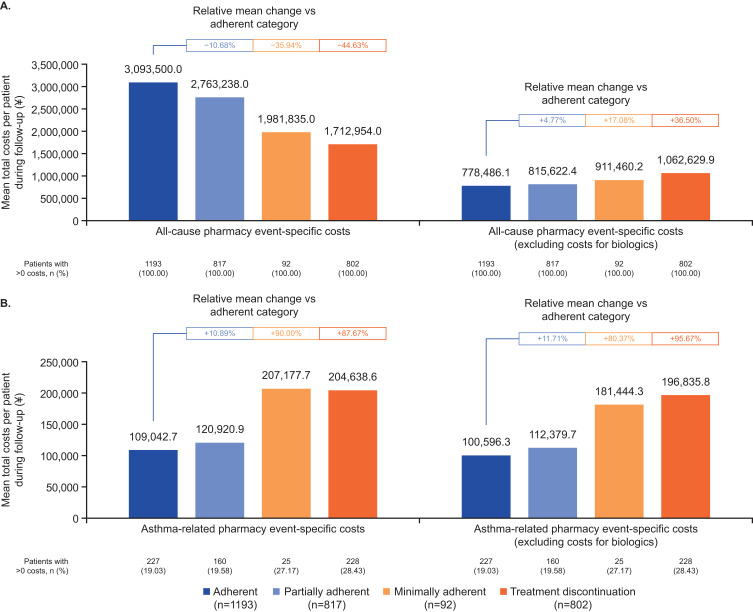
Summary of all-cause (**A**) and asthma-related (**B**) pharmacy costs by adherence categories during the 12-month follow-up period (N=2904).

Event-specific pharmacy costs were stratified by the inclusion or exclusion of biologic therapies. Biologic adherence was generally associated with reduced economic burden, particularly when the costs of biologics were excluded (Supplementary Table 1), consistent with results across adherence categories. When biologics were included in all-cause pharmacy costs, the adherent group had the highest mean expenses (¥3,093,500); costs decreased as adherence declined (Figure 5A). Excluding biologic costs reversed this pattern, with the adherent group incurring the lowest all-cause pharmacy expenses and less adherent groups incurring higher all-cause pharmacy expenses. Asthma-related pharmacy costs, both including and excluding biologics, showed a similar trend, with lowest costs reported in the adherent group (¥109,042.7 and ¥100,596.3, respectively), with costs rising as adherence decreased (Figure 5B).

The analysis of pharmacy costs across the seven GBTM clusters revealed consistent trends that aligned with the adherence categories (Supplementary Figure 3A and B). Patients in higher adherence clusters (notably Cluster A) had greater all-cause pharmacy costs compared with those in lower adherence clusters. However, excluding biologics or focusing on asthma-related pharmacy costs shifted the burden to lower adherence clusters. Clusters F and G were consistently associated with the greatest asthma-related pharmacy costs, with a 96.25%–151.00% relative increase compared with Cluster A.

### Oral and IV Corticosteroid Use

The cumulative and daily average of combined oral and IV corticosteroid use was analyzed by GBTM cluster only (Supplementary Figure 4). The proportion of patients with ≥1 oral and IV corticosteroid use increased with declining adherence from 26.01% in Cluster A to 39.41% in Cluster G. Cumulative oral and IV corticosteroid use exhibited variation across clusters, with the lowest mean doses reported in Clusters A (119.28 mg) and C (117.20 mg), and the highest mean cumulative exposure reported in low adherence Clusters F (283.13 mg) and G (263.37 mg). The mean daily dose followed a similar pattern, with Clusters A–D reporting the lowest daily dose (11.48–13.43 mg) and Cluster G reporting the highest (31.20 mg).

## Discussion

This real-world cross-sectional study used Japan’s Medical Data Vision claims database to explore the relationship between biologic treatment adherence patterns and key clinical and economic outcomes over a 12-month follow-up period in patients with severe asthma in Japan. Adherence patterns were assessed using MPR and GBTM, providing a nuanced view of adherence behavior over time and allowing the identification of distinct adherence categories and clusters, ranging from most to least adherent. Overall, adherence was associated with better clinical outcomes, lower HCRU and reduced pharmacy costs compared with reduced adherence.

Patients who were adherent to their biologic consistently experienced fewer exacerbations requiring hospital admissions, as well as exacerbations requiring ED visits or short-term oral/IV corticosteroids. Biologic adherence was also associated with reduced cumulative oral and IV corticosteroid exposure, which may spare patients from the risk of acute and chronic adverse events, associated with repeat courses of these treatments.^28^ The positive impact of adherence was also observed across the majority of HCRU-related outcomes. These findings align with previous studies, which demonstrate the real-world benefits of asthma biologics in reducing exacerbation frequency, OCS use, and asthma-related hospitalizations in patients with severe asthma in Japan.^9^,^29^,^30^ Although total all-cause pharmacy costs were highest among patients with the greatest adherence, primarily driven by the use of biologics, the trend reversed when biologic costs were excluded or when only asthma-related pharmacy costs were considered, highlighting the true cost-saving effect of adherence in adherent patients. In contrast, patients with lower adherence incurred higher costs, which likely reflects the increased need for additional pharmacological management due to suboptimal asthma control among poorly adherent patients. These findings highlight the critical importance of adherence to biologic treatment, from both a clinical and economic perspective, and emphasize the need to optimize treatment strategies to promote adherence in clinical practice.

The impact of adherence to biologic therapies on clinical and economic outcomes observed in this Japanese population aligns with findings from a similar US-based study (GSK 214570).^31^,^32^ The Japanese cohort demonstrated higher adherence rates compared with those observed in the US cohort, with a greater proportion classified as adherent (41.1% vs 20.0%), and a smaller proportion discontinuing treatment (27.6% vs 54.7%).^31^ These differences may reflect variations in healthcare systems, treatment availability and data sources.

Even though adherence was higher in the Japanese cohort than in the US cohort, our data illustrate that adherence to biologic therapy remains a challenge for some patients in Japan, despite universal national healthcare insurance, where treatment is not restricted by insurance-type. Several factors can impact adherence to biologics in patients with asthma. Shorter dosing intervals, at-home administration, younger age, more frequent exacerbations, emergency visits, or hospitalizations have all been associated with poorer adherence, as these factors can increase the burden of treatment or complicate routine management.^17^,^33–37^ Additionally, psychological barriers to adherence can include fear of injections, depression, denial of illness and stigma.^17^ Poor adherence, in turn, can reduce the effectiveness of therapy, increase the risk of asthma exacerbations, and lead to higher HCRU and overall disease burden, as demonstrated in the current study. Strategies, such as shared decision-making between patients and clinicians, personalized approach to biologic therapy, and the use of biologics with longer dosing intervals may help improve the adherence and reduce treatment burden.

Comparing adherence rates across other studies is challenging due to differences in methodologies, definitions of adherence, and follow-up periods. For example, one US study assessed adherence to five biologics using proportion of days covered (PDC) over six months and found that 61% of patients achieved a PDC ≥0.75.^38^ Another Italian study on omalizumab reported that 90.7% had good adherence over 12 months, defined as missing <10% of doses.^39^ These approaches rely on single-point estimates such as PDC or MPR and, thus, may not fully capture changes in treatment adherence behaviors over time. The use of GBTM in this study offers a more dynamic and comprehensive assessment of treatment adherence patterns; by identifying dynamic trajectories, rather than static measures, GBTM provides a more nuanced understanding of how treatment behaviors evolve over time and their impact on clinical and economic outcomes.

Strengths of this study include its large sample size, the extended observation period (2009–2024), inclusion of data for all five asthma biologics approved in Japan, and the use of GBTM to identify the distinct adherence patterns over time. However, there are some limitations. First, if patients switched to a hospital outside the network, they were lost to follow-up. Second, pharmacy data were not directly linked to medical records, making it difficult to accurately link prescriptions to specific diagnoses or encounters and potentially underestimating asthma-related pharmacy costs. Pharmacy claims also assume that medications are taken as prescribed and may not directly reflect medication intake. Third, as the MDV database does not provide reasons for treatment discontinuation, it is not possible to distinguish between patients who did not receive biologic therapy either due to non-adherence or treatment being stopped by the clinician. Fourth, the study design excluded patients without a full 12 months of follow-up, and those who switched biologics during this period were excluded from the GBTM analyses. While infrequent (only 373 [12.8%] of the 2904 eligible patients switched biologics during the 12-month period of this study), biologic switching does occur in real-world practice and excluding these patients may introduce selection bias and limit the generalizability of the findings by not fully representing real-world treatment complexities. Fifth, patients with comorbid conditions eg atopic dermatitis were excluded, which may also have introduced selection bias. However, this was to ensure that only asthma-related biologic adherence was assessed and to avoid confounding due to differences in biologic dosing schedules between indications. Sixth, as the results could have been influenced by Japan’s healthcare system, caution is necessary when applying them to populations outside Japan. Finally, exacerbation events and HCRU outcomes were limited to hospital admissions, which may have led to underestimation of the total burden. In addition, as with any observational data, causal inferences should be made with caution. The complexity and sensitivity of GBTM could also increase the risk of overfitting. Future studies might consider alternative approaches, such as Latent Class Analysis, to achieve more robust and generalizable classifications. Despite these limitations, the consistency in the results across the treatment adherence categories and the GBTM clusters supports the robustness of the finding in this Japanese cohort. Furthermore, although adherence to biologics may depend on the country’s healthcare system and cost to the patient, the consistency between the findings of the current Japanese cohort and the US cohort study underscores the value of biologic adherence across diverse populations.

## Conclusion

In conclusion, this study demonstrates that adherence to asthma biologic therapies is associated with fewer exacerbations, lower HCRU, and decreased asthma-related pharmacy costs compared with reduced adherence in patients with severe asthma in Japan. These findings provide robust evidence to inform clinical and policy decisions in Japan and contribute to the growing body of international evidence supporting the real-world value of adherence to asthma biologic treatment in patients with severe asthma.

## Data Availability

Data are owned by the Medical Data Vision database, managed by Medical Data Vision Co., Ltd and were accessed by GSK to address the prespecified research questions only.

## Abbreviations

BEC, blood eosinophil count; BMI, body mass index; ED, emergency department; GBTM, group-based trajectory modeling; GLMM, generalized linear mixed mo HCRU, healthcare resource utilization; ICS, inhaled corticosteroid; IgE, immunoglobulin E; IL, interleukin; IQR, interquartile range; IV, intravenous; LABA, long-acting beta-2 agonist; LAMA, long-acting muscarinic antagonists; MPR, medication possession ratio; OCS, oral corticosteroid; PDC, proportion of days covered; Q2W, every 2 weeks; Q4W, every 4 weeks; Q8W, every 8 weeks; SD, standard deviation; SMD, standardized mean differences.
